# A Porcine-Derived Heme Iron Powder Restores Hemoglobin in Anemic Rats

**DOI:** 10.3390/nu16234029

**Published:** 2024-11-25

**Authors:** James H. Swain, Logan D. Glosser

**Affiliations:** 1 Department of Nutrition, School of Medicine, Case Western Reserve University, 10900 Euclid Avenue, Cleveland, OH 44106, USA; 2 School of Medicine, Emory University, 100 Woodruff Circle, Atlanta, GA 30322, USA

**Keywords:** heme iron, iron absorption, hemoglobin regeneration efficiency, micronutrient, food fortification

## Abstract

Background/Objectives: Iron-fortified foods reduce the incidence of iron deficiency anemia. However, the nutritional efficacy of heme iron fortificants is unclear. Methods: In this study, we determined the hemoglobin regeneration efficiency (HRE) of a porcine-derived heme iron powder (HIP), treating anemic rats (hemoglobin (Hb) 3–6 g/dL) with 14-day repletion diets fortified with four different concentrations (12, 24, 36, or 48 mg iron/kg diets) of HIP or a control diet (“no added iron”); *n* = 9–12/group. Results: Our results demonstrate an inverse association between HRE and increasing dietary iron from the HIP. The HRE ratios of diets containing the HIP powder at 12, 24, 36, or 48 mg iron/kg were 0.508, 0.268, 0.273, and 0.223, respectively. Based on the mean final Hb values at 14 d, the HRE ratio of the 12 mg iron/kg diet was significantly higher (*p* ≤ 0.05) compared to the other HIP diet groups; however, only the HIP provided in the 36 and 48 mg iron/kg diets restored hemoglobin to high enough levels (mean Hb > 6 g/dL) to correct anemia. Conclusions: Our findings show that HIP at each of the concentrations tested increased Hb; moreover, when present at higher concentrations in the diet, the HIP is capable of restoring hemoglobin to resolve iron deficiency anemia.

## 1. Introduction

Iron deficiency anemia (IDA) is the most prevalent micronutrient deficiency among humans worldwide [1,2,3]. IDA results in significant reductions in work productivity and adds billions of dollars to the cost of health care, burdening already stressed medical systems [4]. To correct IDA, staple foods, especially cereal grain flours, are fortified with different forms of iron, including elemental and heme sources [5,6,7,8]. However, the efficacy of many types of iron fortificants is unclear [9,10,11,12,13,14,15].

Increasingly, there is interest in the use of bovine- and porcine-derived heme iron powders (HIPs) to serve as fortificants in a variety of foods since iron in the heme form has relatively good bioavailability and is less influenced by inhibitors of non-heme iron absorption, such as tannins, phytic acid, and calcium, which greatly reduce iron absorption [16,17,18,19]. Heme iron is also more highly absorbed and better tolerated (less gastrointestinal discomfort) when compared to non-heme iron powder intake; iron from HIPs also results in less oxidative stress intraluminally [9,11,13,19,20]. HIPs are polypeptides that contain a soluble heme moiety derived from the enzymatic digestion of bovine or porcine hemoglobin [7,19]. The bioavailability of iron from HIPs has been found to be 40–60% higher than iron from non-heme elemental iron powders or iron salts, such as ferrous sulfate [10,20,21]. Although heme iron intake contributes greatly to the overall iron absorption within a well-balanced, omnivorous (meat-containing) diet, a better understanding of its absorption from commercially prepared heme iron powder fortificants may assist in developing enhanced dietary guidelines [21,22].

Iron absorption, bioavailability, and the impact of different forms of dietary iron on hemoglobin and iron status have been investigated in murine and avian models, as well as in humans [23,24,25,26,27]. Among murine models, the rat hemoglobin repletion assay has been shown to be an appropriate approach for studying iron intake and absorption, hemoglobin response, and resolution of IDA using different forms of dietary iron [23,25,26,27,28].

The purpose of this study was to determine the hemoglobin regeneration efficiency (HRE) of a heme iron powder (HIP) by determining the change in hemoglobin and hemoglobin iron in anemic rats as they consumed graded (increased) quantities of HIP during a 14-day iron repletion period. The aim was to better understand the absorption of iron from the HIP and thereby help develop more specific dietary guidelines regarding the use of HIPs as alternative food fortification approaches.

## 2. Materials and Methods

### 2.1. Heme Iron Powder

This study tested a heme iron powder (HIP) (Proliant, Inc., Ankeny, IA, USA/APC Europe, S.A., Barcelona, Spain). A “no added iron” diet served as the control. The HIP was spray-dried blood in powdered form (porcine origin), dark brown/black in color, similar to that described previously [7]. The HIP used in this study had the following characteristics: 78.13% protein, 9.57% ash, 1.48% iron, and 5.6% humidity. The HIP was subjected to 80 °C for at least 2 h during production. Upon receipt, the HIP was stored in a desiccator under vacuum and refrigerated (3 °C) until use.

### 2.2. Study Design and Dietary Treatments

This study initially used 72 male weanling Sprague Dawley rats (Charles River/SASCO, Wilmington, MA, USA). The rats were housed individually in stainless steel mesh wire-bottom cages at 21 ± 1 °C and provided with a 12-h light–dark cycle. After a 24-day depletion period consuming an iron-deficient diet (1.6 mg iron/kg AIN-93G[M] diet; approximately 1.4 mg iron/kg diet-analyzed iron content), anemic rats with hemoglobin values between 3 and 6 g/dL (mean ± SEM of 3.9 ± 0.6 g/dL; range 3.1–5.8 g/dL) were then randomly assigned to one of five different iron repletion-period diet groups, with the blocking based on hemoglobin. The rats were then provided with repletion diets for 14 d, fortified with HIP or a control diet (“no added iron”); *n* = 9–12/group. All diets and deionized, distilled water were provided ad libitum. The HRE ratio calculation accounts for body weight and iron intake; therefore, during the repletion period, animal weight and food consumption measurements were performed daily, including adjustments for spilled food, to determine both daily and total iron intake. All animal procedures followed the Institutional Animal Care and Use Committee (IACUC) procedures at Case Western Reserve University (CWRU) in accordance with NIH guidelines.

The HIP was incorporated into diets modified to have a very low base iron content, using vitamin-free casein (Harlan Teklad, Madison, WI, USA), a modified mineral mix that omitted ferric citrate (Harlan Teklad), a high purity microcrystalline cellulose fiber source (Alphacel™; ICN Biomedicals, Irvine, CA, USA), and reagent grade ingredients. The diets were also phytate-free, with a neutral pH (7.0). The baseline-modified diet [29] (AIN-93G[M]) composition, from which treatment (repletion-period) diets were prepared, is shown in Table 1. Without added iron, the diet contained approximately 1.4 mg iron/kg by analysis. To prepare repletion-period diets, the HIP was added, taking into account the baseline amount (analyzed) already present in the control (“no added iron”) group.

Mixing of the HIP into repletion-period diets was performed as previously described [5]. The HIP contained 1.48% iron (*w*/*w*); the iron concentration present in the HIP was used to determine the amounts added to the baseline diet to attain the desired iron concentrations in treatment diets. Inductively coupled plasma optical emission spectroscopy (ICP-OES; Series 720/730; Agilent, Inc., Santa Clara, CA, USA) was used to confirm the iron concentration in the HIP. Thereafter, prior to the repletion period, small portions of each repletion-period treatment diet and the control (“no added iron”) diet were also taken for analysis to confirm the iron content as previously described [19].

### 2.3. Hemoglobin and Hemoglobin Iron Determinations

Procedures for determining hemoglobin (Hb) and Hb iron, phlebotomy, and animal sacrifice following anesthetization were conducted as previously described [5]. Briefly, the following calculation was used to determined Hb iron:

Hb Fe (mg) = BW (kg) × 0.067 × Grams Hb per mL × 3.35 mg Fe

It is important to note that the calculation assumes that the blood is 6.7% body weight (BW; kg) and hemoglobin iron content is 3.35 mg/g [8,26]. Therefore, the Hb iron was determined on the basis of 3.35 mg iron/g Hb and 0.075 L blood/kg body weight [23,28].

### 2.4. Hemoglobin Regeneration Efficiency

The hemoglobin (Hb) regeneration efficiency (HRE) of the HIP was determined as described previously [5]. Briefly, HRE ratios were calculated using the analyzed value of iron for each diet based on the following formula:

HRE ratio = [Final Hb Fe (mg) − Initial Hb Fe (mg)]/Fe intake (mg total consumed; analyzed diet value)

### 2.5. Statistical Analyses

A preliminary power analysis was conducted based on previously published data [5,13,23]. Statistical analyses of hematological indices and hemoglobin (Hb) repletion data were performed as described previously [5,23,28,30,31,32]. The control diet (“no added iron”) group served as a point of reference for comparing the Hb increase from the baseline diet. Differences between the diet group mean values, including for HRE, were tested using Tukey’s multiple comparison test and Duncan post-hoc testing using the statistical package SAS (SAS Version 10.2, SAS Institute, Cary, NC, USA). Data illustrations were performed using GraphPad Prism (Software version 10.2; GraphPad, Boston, MA, USA). Values were expressed as mean values ± SEM. Significance was set at *p* ≤ 0.05.

## 3. Results

### 3.1. Hemoglobin and Hemoglobin Iron Change

**Figure 1:**
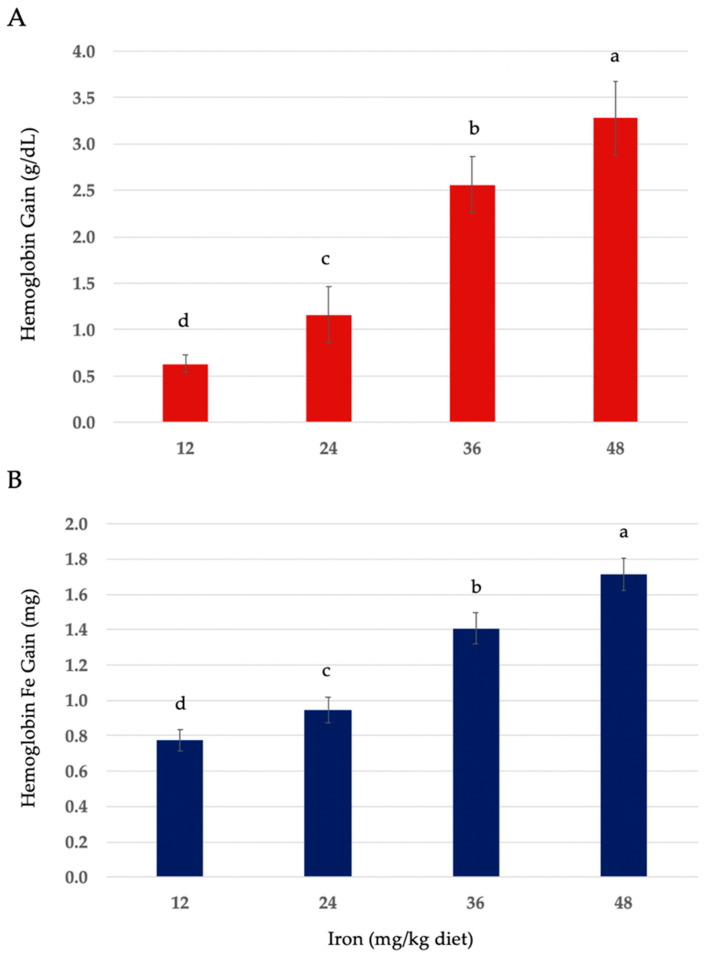
(**A**) Hemoglobin (Hb) gain (g/dL). (**B**) Hemoglobin iron (Fe) gain in anemic rats fed 12, 24, 36, and 48 mg iron/kg diets in the form of heme iron powder for a 14-day repletion period. Values are mean values ± SEM (*n* = 9–12/group). Different letters (a–d) are used to denote significant differences (*p* ≤ 0.05) from higher to lower hemoglobin and hemoglobin Fe gain. Hb Fe (mg) = BW (body weight; kg) × 0.067 × Grams Hb per mL × 3.35 mg Fe.

The hematological values of anemic rats fed the control diet (“no added iron”) or on the 12, 24, 36, and 48 mg iron/kg diets, with iron added in the form of heme iron powder (HIP), are shown in Table 2. Food intake and weight gain were positively associated with increasing dietary iron in all the treatment groups (Table 2). Iron intake (mg/day) was positively associated with dietary iron concentration (Figure S1). Hemoglobin changes and hemoglobin iron (Fe) gain in anemic rats fed HIP were positively associated with iron concentration in the diet (Figure 1A,B).

### 3.2. Hemoglobin Regeneration Efficiency of Heme Iron Powder

**Figure 2:**
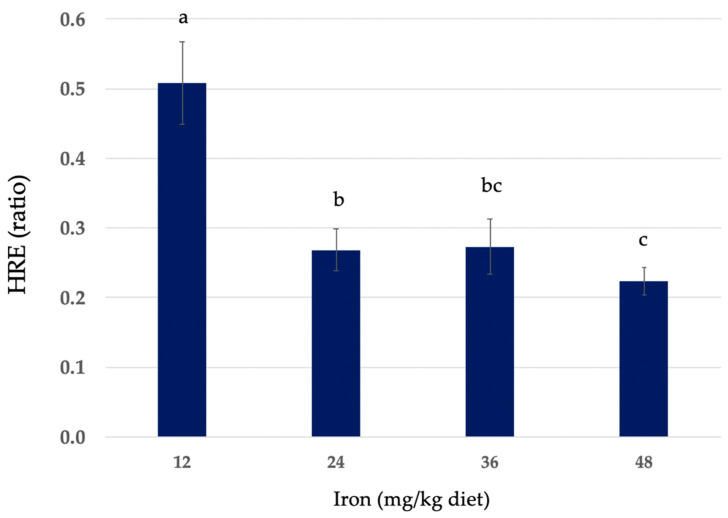
Hemoglobin (Hb) regeneration efficiency (HRE) in anemic rats fed 12, 24, 36, and 48 mg iron/kg diets in the form of heme iron powder for a 14-day repletion period. Values are mean values ± SEM (*n* = 9–12/group). Different letters (a–c) are used to denote significant differences (*p* ≤ 0.05) from higher to lower HRE. HRE ratio = [Final Hb Fe (mg) − Initial Hb Fe (mg)]/Fe intake (mg total consumed).

Hemoglobin regeneration efficiency (HRE) ratios of diets containing HIP are also shown in Table 2. The HRE ratio calculation accounts for body weight and iron intake. The HRE ratios of HIP diets containing 12, 24, 36, and 48 mg iron/kg expressed as mean values ± SEM were 0.508 ± 0.06, 0.268 ± 0.03, 0.273 ± 0.04, and 0.223 ± 0.03, respectively. The comparative HRE ratios of the HIP at each level of dietary iron are shown in Figure 2.

## 4. Discussion

Determining the hemoglobin regeneration efficiency using the rat hemoglobin repletion assay has been identified by other research groups as a proficient and valid approach to test the ability of different forms of dietary iron to resolve the anemia of iron deficiency [23,24,28,30,31,32,33,34,35]. Findings from this study are valuable by demonstrating that iron from this type of heme iron powder (HIP) increased hemoglobin at each of the concentrations tested and that at higher concentrations in the diet (36 and 48 mg iron/kg), the HIP is capable of restoring hemoglobin to resolve iron deficiency anemia (IDA). Our data are also beneficial in illustrating the HRE ratios of the HIP at four different concentrations of iron tested in a diet with minimal (negligible) background iron.

Findings from this study show that the hemoglobin change and hemoglobin iron gain in anemic rats fed HIP were positively associated with iron concentration in the diet. Our results are in agreement with the findings of other studies that have investigated the hematological effects of different forms of iron powders [26,27,28,30,34,35]. Overall, we found that HRE was inversely associated with increasing dietary iron. Although the HRE ratio of the diet containing the lowest iron concentration (12 mg iron/kg diet) was significantly higher (*p* ≤ 0.05) than the other HIP diet groups based on the mean final hemoglobin at 14 d, only the HIP provided at the two greater concentrations of iron tested in this study (36 and 48 mg iron/kg diets) restored hemoglobin to adequate levels to correct anemia (Hb > 6 g/dL). A prior study on the effect of dietary iron levels on the efficiency of converting non-heme iron into hemoglobin in anemic rats found that the efficiency of conversion of dietary iron into hemoglobin iron was not significantly affected by the level of dietary iron [35]. These results are in contrast to our results, which demonstrated that at lower levels, HRE was significantly higher (*p* ≤ 0.05). Thus, our findings show a greater proportional gain in hemoglobin with less heme iron consumed; as the amount of dietary iron from the HIP increased, the proportional gain in hemoglobin was less. This result is similar to the findings of previous studies that investigated the total percentage of dietary iron absorption [32,36].

A study investigating the bioavailability of iron from fresh, cooked, or nitrosylated hemoglobin to anemic rats found similar, albeit slightly lower, HRE values for their hemoglobin product [34], especially when compared to the HRE values we obtained at the 12 mg iron/kg diet level. This study also found that the efficiency of hemoglobin regeneration in anemic rats fed nitrosylated hemoglobin was lower compared to unnitrosylated products and that cooking, as in our porcine sample, did not affect the availability of the heme iron [34]. The influence of other dietary inhibitors and enhancers of iron absorption, especially affecting non-heme iron absorption, has been well studied, including extensive reviews [37,38]. In countries with high meat consumption, heme iron may comprise one-third of total dietary iron yet account for two-thirds of the iron absorbed by the body, attributed to the selective preference for heme iron absorption, since it remains soluble in the small intestine, and because heme iron absorption by enterocytes is not adversely affected by dietary inhibitors [37,38]. Therefore, the use of HIP as a food fortificant may favorably overcome the dietary inhibition caused by phytate, tannins, and calcium, which is often observed when testing the bioavailability of inorganic iron. 

One limitation of this study may be that additional concentrations of dietary iron from the HIP (that is, <12 or >48 mg iron/kg diets) were not used. However, our study design, which included four distinct graded (increasing) concentrations of dietary iron, reflects a design approach that has been used to test a variety of other forms of iron fortificants. Hence, the four iron concentrations used in this study were used as a framework for comparing our results to other studies in the field of iron absorption and assessment of HRE using murine models. Another limitation may be that we used rats, whereas some studies used mice or species other than rodents. However, because most other animal models of human iron absorption have been murine models, especially rats, this study was designed to enable further comparisons to previous research using the hemoglobin repletion assay; it is important to note that this assay typically uses a threshold for selecting rats for repletion treatment set at a lower level of anemia, considering that in humans a value of 6 g/dl Hb is the threshold for blood transfusion and anemia is usually assumed at values <10 g/dL. Further, the measurement of HRE as part of this assay may not take into account the effects of iron overload, especially at higher levels of iron in the diet. Nevertheless, other studies have found that the efficiency of converting dietary iron into hemoglobin by anemic rats was very similar to reported absorption values for heme iron by iron-deficient human subjects [34,37,38,39]. Therefore, data from this study may be useful when considering new food fortification policies and guidelines and the potential utilization of heme iron powders as part of global micronutrient fortification programs under consideration [22]. Although serum transferrin, ferritin, and total iron binding capacity (TIBC) were not measured because the focus of this study was HRE, understanding how these indices change in the context of hemoglobin repletion would provide beneficial insights. Additionally, because of religious or vegetarian dietary practices, this HIP may not be suitable or accepted as a food fortificant.

Overall, data from this study are valuable in demonstrating that the absorption of iron from this particular HIP increases hemoglobin and thereby reduces IDA. Anemia is generally considered to be a symptom rather than a disease because a decrease in the number of red blood cells and erythrocytic hemoglobin (mean cell hemoglobin content) can be caused by nutritional deficiencies or a variety of underlying medical conditions, such as bleeding disorders and chronic diseases, with thalassemia or chronic kidney diseases, respectively, common non-nutritional etiologies. Therefore, identifying the etiology of anemia is vital for treatment. Because current food fortification guidelines for iron, especially using non-heme elemental iron or iron salts and fortification of staple foods (grain flours), suggest varying the amount of iron used based on the type or form of iron, our findings are also beneficial in illustrating the usefulness of HIP as an alternative food fortification approach that may be effective in reducing IDA. Using the HIP as a dietary supplement may also be beneficial in preventing and/or minimizing IDA, reducing the need for clinical care, and thereby helping to reduce medical costs. Although dietary therapy for anemia is a well-established approach and iron supplements may be purchased inexpensively, the use of heme iron powder offers an effective alternative approach and another efficacious option to acquire and consume a form of relatively well-absorbed dietary iron and thereby help prevent IDA.

Globally, iron deficiency anemia remains one of the most common micronutrient deficiencies [40,41,42]. A recommendation to help combat persistent IDA in communities worldwide is for additional studies of HIP to include human clinical trials to determine optimal levels of HIP in the diet, providing hematological support to individuals at risk of developing IDA.

## 5. Conclusions

In summary, our findings are valuable for illustrating that this heme iron powder is a useful fortification agent to replenish hemoglobin and resolve iron deficiency anemia. Considering the favorable absorption profile of heme in comparison to non-heme iron, the inclusion of heme iron powder as an iron fortifcant in a variety of staple foods may be both efficacious and advantageous in reducing the incidence of iron deficiency and its associated anemia.

## Figures and Tables

**Table 1:** Treatment diets were prepared by adding iron (Fe) as heme iron powder (HIP) to the following baseline diet composition ^1^.

Formula		g/Kg
Corn Starch		397.5
Casein ^2^		200
Maltodextrin		132
Sucrose		100
Soybean Oil		70
Cellulose, microcrystalline (Alphacel™)		50
Mineral Mix modified, no added iron (06053)		35
Vitamin Mix, AIN-93-VX (94047) ^3^		10
L-Cystine		3
Choline Bitartrate		2.5
TBHQ, antioxidant ^4^		0.014
Macronutrient	% Dry Weight	% Kcal
Protein	18.3	19.4
Carbohydrate	60.1	63.8
Fat	7	16.7

^1^ Ref: [29]. ^2^ Alcohol-extracted, vitamin-free, casein. ^3^ Ascorbic acid at 200 mg/kg diet. ^4^ Tertiary-butylhydroquinone. Catalog numbers are shown in parentheses for mineral and vitamin mixes at the time of preparation. All diet ingredients were obtained from Harlan Teklad, Madison, WI, USA.

**Table 2:** Food and iron (Fe) intake, growth, hemoglobin Fe change, and hemoglobin regeneration efficiency (HRE) in anemic rats fed graded quantities of the heme iron powder (HIP) for a 14-day repletion period ^1,2^.

	Control (“No Added Iron”)	Heme Iron Powder (HIP)			
Diet Code	C	HIP-1	HIP-2	HIP-3	HIP-4
Diet Fe (mg/kg)					
Calculated	1.6	12	24	36	48
(Analyzed)	(1.4)	(11.6)	(25.3)	(34.2)	(47.1)
Food intake (g/day)	11.7 ± 0.59 ^d^	13.2 ± 0.64 ^c^	13.9 ± 0.67 ^bc^	15.1 ± 0.85 ^ab^	16.3 ± 0.90 ^a^
Fe intake (mg/day)	0.016 ± 7^−4 e^	0.153 ± 0.02 ^d^	0.352 ± 0.06 ^c^	0.516 ± 0.06 ^b^	0.768 ± 0.09 ^a^
Body weight (g)					
Initial	83.9 ± 3.8 ^a^	84.1 ± 3.6 ^a^	83.6 ± 3.5 ^a^	83.2 ± 3.8 ^a^	85.5 ± 3.7 ^a^
(Gain)	(15.2 ± 0.9 ^c^)	(53.8 ± 2.9 ^b^)	(54.9 ± 3.0 ^ab^)	(56.3 ± 3.4 ^ab^)	(59.4 ± 3.5 ^a^)
Hemoglobin (g/dL)					
Initial	4.63 ± 0.5 ^a^	4.82 ± 0.7 ^a^	4.73 ± 0.5 ^a^	4.79 ± 0.6 ^a^	4.85 ± 0.9 ^a^
(Gain)	(−0.42 ± 0.04 ^e^)	(0.63 ± 0.1 ^d^)	(1.16 ± 0.3 ^c^)	(2.56 ± 0.3 ^b^)	(3.28 ± 0.4 ^a^)
Final	4.21 ± 0.4 ^c^	5.45 ± 0.7 ^b^	5.89 ± 0.9 ^b^	7.35 ± 1.1 ^a^	8.13 ± 1.3 ^a^
Hemoglobin Fe ^3^					
Gain (mg)	0.064 ± 8^−3 e^	0.777 ± 0.06 ^d^	0.944 ± 0.07 ^c^	1.407 ± 0.09 ^b^	1.713 ± 0.09 ^a^
HRE ^4,5^	-	0.508 ± 0.06 ^a^	0.268 ± 0.03 ^b^	0.273 ± 0.04 ^bc^	0.223 ± 0.02 ^c^

^1^ Values are mean values ± SEM (*n* = 9–12/group). ^2^ Different letters (a–e) are used to denote significant differences (*p* ≤ 0.05) from higher to lower mean values within a row. ^3^ Hb Fe (mg) = BW (body weight; kg) × 0.067 × Grams Hb per mL × 3.35 mg Fe. ^4^ HRE ratio = [Final Hb Fe (mg) − Initial Hb Fe (mg)]/Fe intake (mg total consumed). ^5^ A dash mark in the “No Added Iron” column for HRE indicates it is not applicable since there was no Hb increase in the control.

## Data Availability

The original contributions and data on which findings are presented in this study are included within the article, as well as the supporting material that accompanies this submission.

## Supplementary Materials

The following supporting information can be downloaded at: https://www.mdpi.com/article/10.3390/nu16234029/s1, Figure S1: Iron (Fe) intake (mg/day) of anemic rats fed graded quantities of heme iron powder for a 14-day repletion period. Values are mean values ± SEM (*n* = 9–12/group). Different letters are used to denote significant differences (*p* ≤ 0.05) from higher to lower Fe intakes.
