# Supplementary material for: A Porcine-Derived Heme Iron Powder Restores Hemoglobin in Anemic Rats
Source: Nutrients. 2024 Nov 25;16(23):4029. doi: 10.3390/nu16234029 (PMC11643988; doi:10.3390/nu16234029)
Supplement: Supplementary file 1 [file nutrients-16-04029-s001.zip › nutrients-3260373-supplementary.pdf]

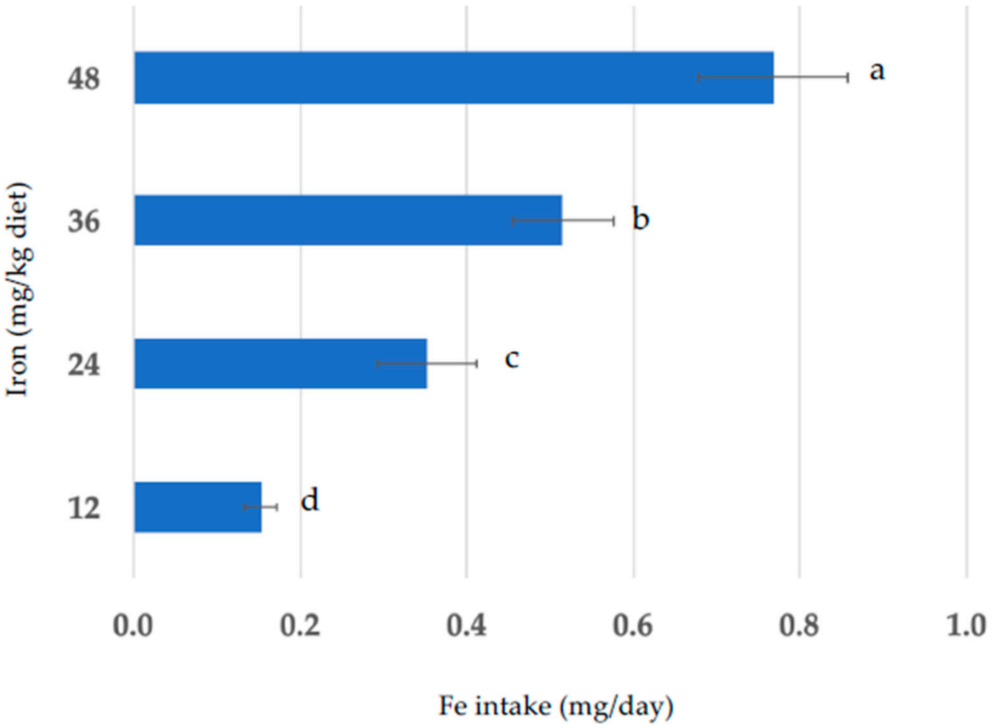

Fig. 1S. Iron (Fe) intake (mg/day) of anemic rats fed graded quantities of heme iron powder for a 14-day repletion period. Values are mean ± SEM (n=9-12/group). Different letters are used to denote significant differences ($p \leq 0.05$), from higher to lower iron intake.
